# Case Series on Endogenous Klebsiella pneumoniae Endophthalmitis: More Than Meets the Eye

**DOI:** 10.7759/cureus.15929

**Published:** 2021-06-25

**Authors:** Pavitra Danapal, Mushawiahti Mustapha, Nur Syarafina Abdul Malek, Justin Yeak, Fazilawati A Qamarruddin

**Affiliations:** 1 Department of Ophthalmology, Pusat Perubatan Universiti Kebangsaan Malaysia, Kuala Lumpur, MYS; 2 Department of Ophthalmology, Hospital Tengku Ampuan Rahimah, Klang, MYS

**Keywords:** endogenous klebsiella endophthalmitis, sight and life threatening, prompt diagnosis, improve survival and visual prognosis, klebsiella pneumoniae invasive syndrome

## Abstract

Endogenous endophthalmitis (EE) is a rare but potentially sight-threatening disease with an appreciable mortality rate. Diabetes mellitus remains the most frequently associated condition especially in the Asian population, which potentiates *Klebsiella pneumoniae* involvement. Endogenous *Klebsiella pneumoniae* endophthalmitis (EKE) usually has a poor final visual outcome despite treatment with intravitreal and systemic antibiotics. We report three cases of EKE with systemic involvement *Klebsiella pneumoniae* invasive syndrome (KPIS).

KPIS was diagnosed in three patients with multiple comorbidities who presented with a blurring of vision and eye redness. Patient 1 was a 63-year-old Malay man diagnosed with left eye panophthalmitis with multifocal liver and prostate abscesses. He underwent drainage of the liver abscess and eventually evisceration of the left eye due to scleral perforation. Patient 2 was a 66-year-old Malay woman diagnosed with left eye endophthalmitis. Due to hemodynamic instability, vitrectomy was delayed and eventually sustained corneal perforation and eviscerated. The patient eventually succumbed to infection. Patient 3 was a 42-year-old Malay woman diagnosed with KPIS, renal abscess, lung abscess, and left endogenous endophthalmitis. She underwent a vitrectomy but her postoperative vision remained poor. All patients received multiple intravitreal antibiotics and systemic antibiotics.

KPIS is frequently associated with catastrophic disabilities. Our cases highlight the importance of an early suspicion of systemic involvement in patients presenting with EKE. Prompt diagnosis, emergent radiographic evaluation, early adequate drainage, and appropriate treatment with antibiotics potentially improve survival and visual prognosis.

## Introduction

Endogenous endophthalmitis (EE) is a rare but potentially sight-threatening disease with an appreciable mortality rate [1-2]. It is a sequel of hematogenous spread from a distant source that breaches the blood ocular barrier causing infection of the intraocular tissue [2]. A gram-negative organism, particularly *Klebsiella pneumoniae,* is the most common causative organism for EE among the Asian population, particularly the East Asians [2-3]. Endogenous *Klebsiella pneumoniae* endophthalmitis (EKE) usually has a poor final visual outcome despite treatment with intravitreal and systemic antibiotics [4-5].

We herein report three cases of EKE in which visual acuity (VA) did not improve after treatment. We discuss the clinical characteristics and treatment outcomes of each patient.

## Case presentation

Case 1

**Figure 1:**
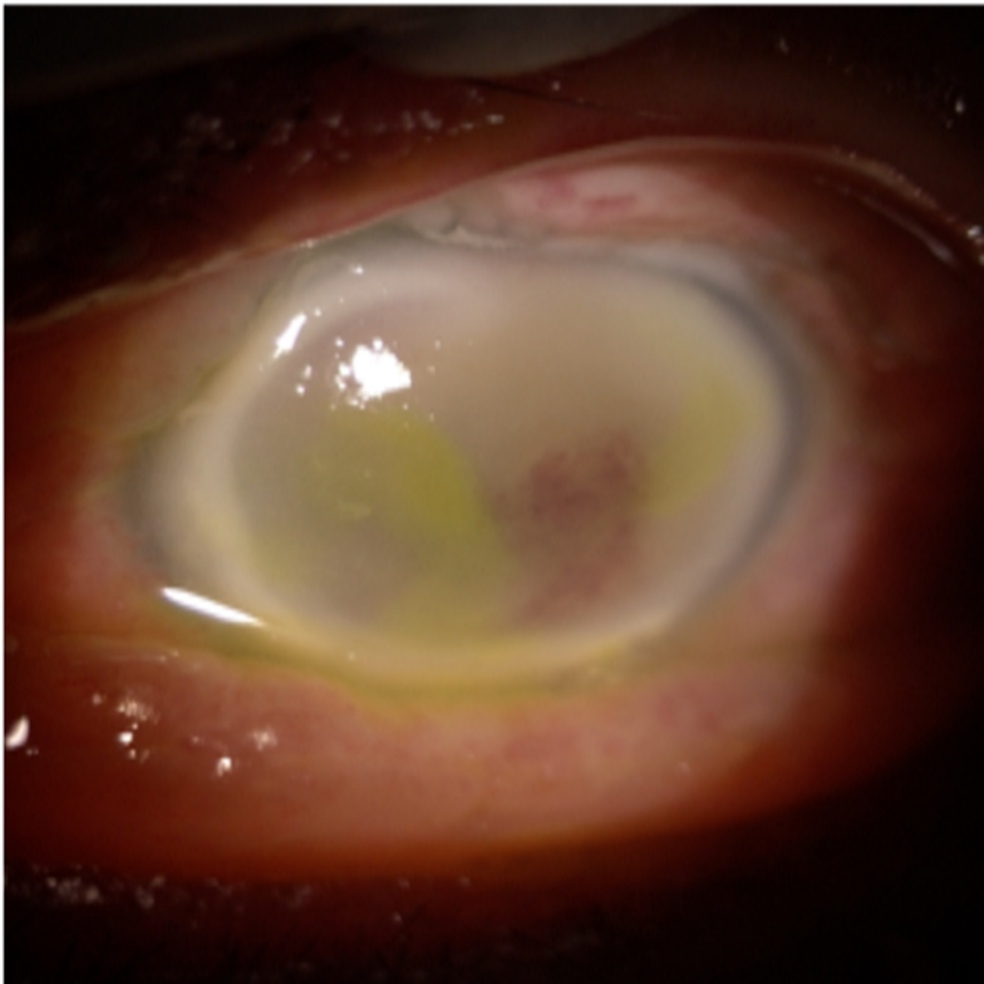
Left anterior segment at presentation showing chemosis, oedematous cornea, and fibrin

A 63-year-old Malay gentleman with underlying diabetes mellitus, hypertension, and chronic kidney disease, presented with a one-week history of left eye blurring of vision, redness, and fever. Upon presentation, visual acuity was 6/12 in the right eye and light perception (PL) in the left eye with positive relative afferent pupillary defect (RAPD). The left eye was chemosed with oedematous cornea and fibrin in the anterior chamber (Figure 1). Intraocular pressure (IOP) was 44 mmHg.

**Figure 2:**
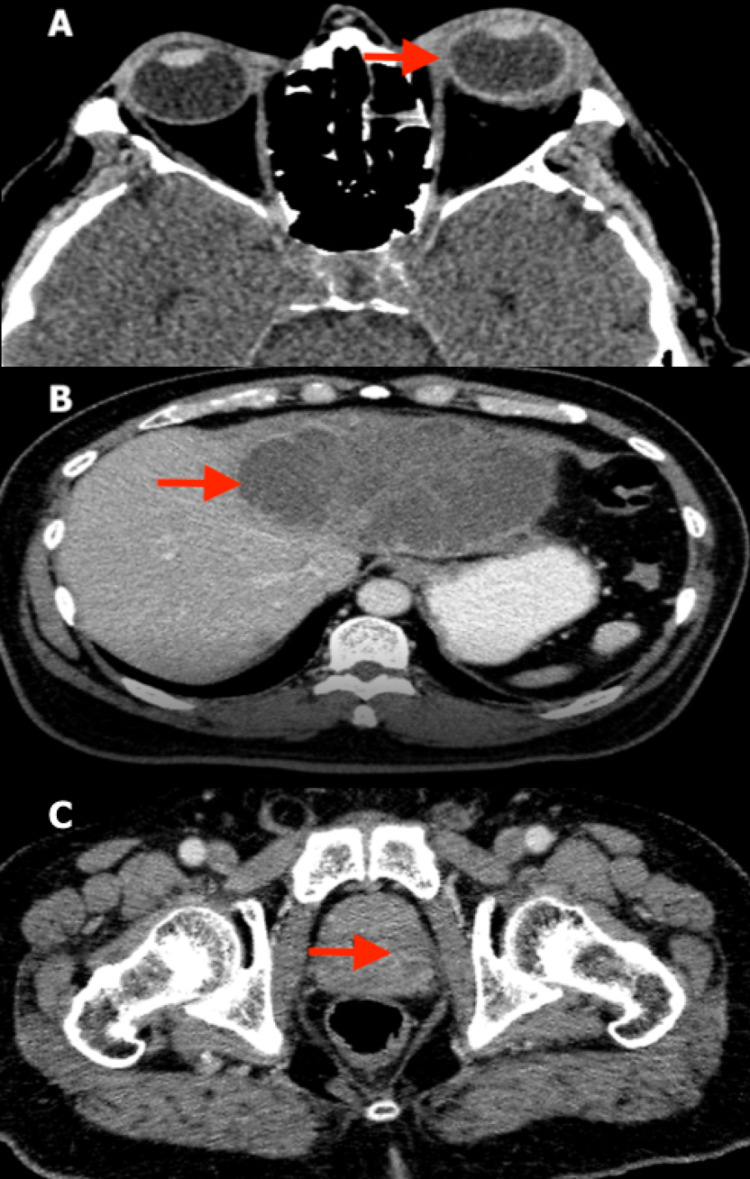
Contrast-enhanced computed tomography (CECT) scans (A) CECT orbit shows left proptosis with thickening and enhancement of the sclera. There is also enhancement of the periorbital soft tissue. Overall findings suggest left panophthalmitis. (B) CECT abdomen shows multiloculated rim-enhancing hypodense collection involving mostly the left lobe of the liver suggestive of liver abscess. (C) CECT pelvis shows hypo enhancement in the left lobe of the prostate suggestive of an abscess.

There was no view of the left fundus. An ultrasound scan of the left eye showed the presence of loculations. Besides having a high-grade fever of 38 degrees Celsius, other systemic examinations were unremarkable. Blood culture and sensitivity yielded *Klebsiella pneumoniae.* A computed tomography (CT) scan of the orbit showed left proptosis and thickening and enhancement of the sclera, with an enhancement of the periorbital soft tissue suggestive of left panophthalmitis. Contrast-enhanced computed tomography (CECT) abdomen/pelvis showed multifocal abscesses in the liver and left prostate (Figure 2). He was treated for KPIS.

Multiple vitreous taps and intravitreal injections (vancomycin 1 mg/0.1 ml and ceftazidime 2.25 mg/0.1 ml) were performed. *Klebsiella pneumoniae* was detected from vitreous and liver tissue cultures. Systemic ceftriaxone, metronidazole, and fortified topical antibiotics were commenced.

**Figure 3:**
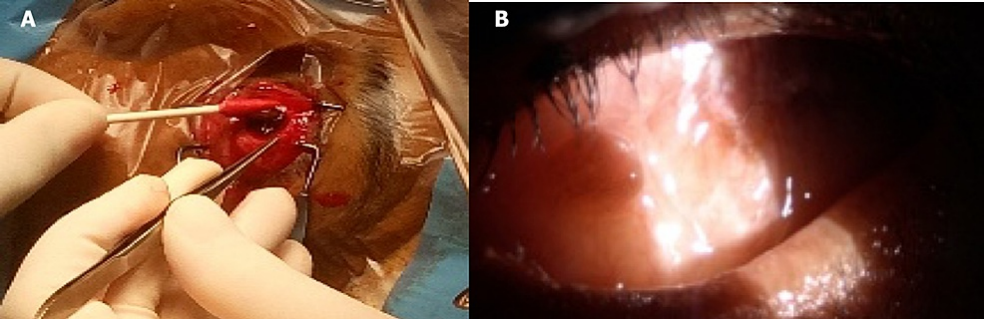
(A) Process of evisceration and (B) Post evisceration

He underwent percutaneous drainage and a biopsy of the liver abscess. Subsequently, a left vitrectomy was done. However, one week later, his left eye was complicated with scleral perforation and underwent evisceration (Figure 3).

Case 2

A 66-year-old Malay woman with underlying diabetes mellitus, hypertension, rectosigmoid carcinoma, and chronic liver cirrhosis presented to the hospital with a three-day history of left eye redness, blurring of vision, and intermittent fever.

**Figure 4:**
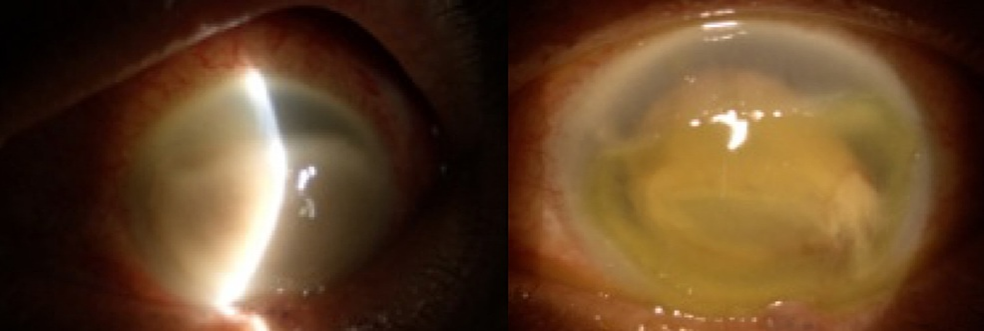
Injected conjunctiva, oedematous cornea, and fibrin with subtotal cornea epithelial defect

Vision in the right eye was 6/12 and hand movement (HM) in the left eye. RAPD was negative. Left conjunctiva was injected, the cornea was oedematous, and the anterior chamber was deep with cells 4+ and fibrin (Figure 4). There was no view of the left fundus.

**Figure 5:**
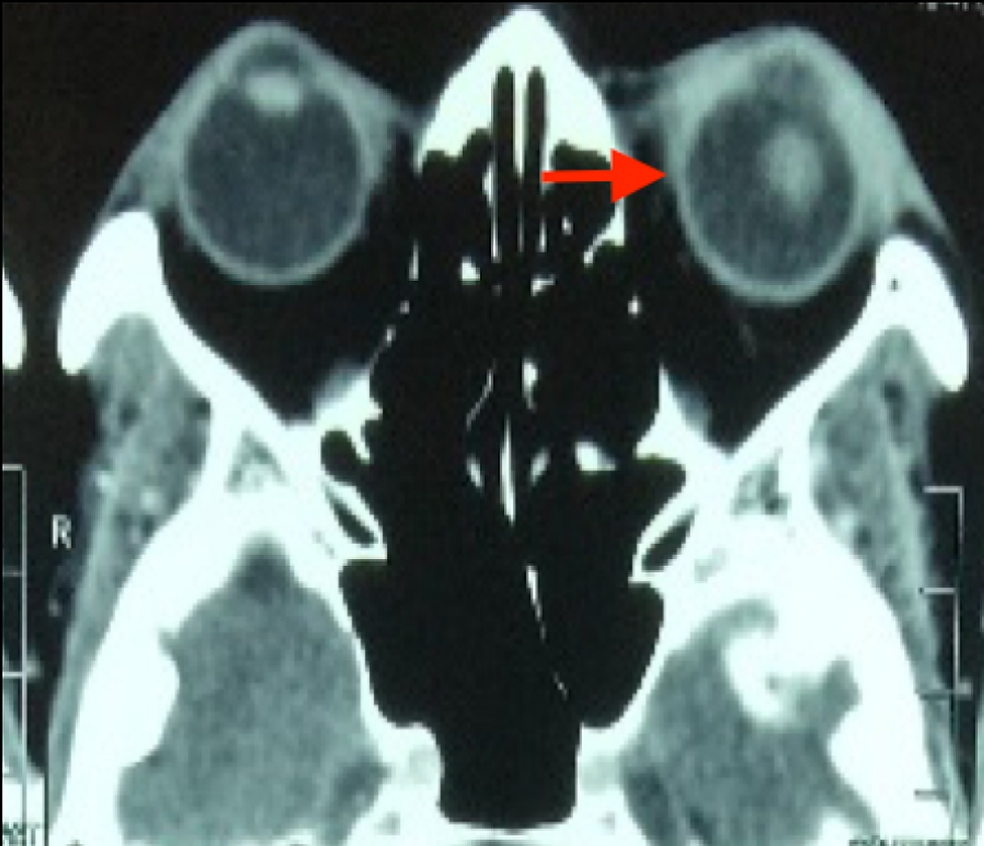
CECT orbit shows left periorbital soft tissue thickening and enhancement suggestive of inflammatory/infective changes. There is also lens dislocation probably due to the breakdown of zonular fibers by Klebsiella pneumoniae. CECT: contrast-enhanced computed tomography

An ultrasound scan of the left eye revealed vitritis with loculation. CECT brain and orbit showed left periorbital soft tissue thickening and enhancement suggestive of inflammatory/infective changes. There was also lens dislocation noted (Figure 5). Abdominal ultrasonography (USG) showed liver cirrhosis but no abscess was detected.

A vitreous tap of the left eye yielded *Klebsiella pneumoniae*. Multiple intravitreal antibiotic injections (vancomycin 1 mg/0.1 ml and ceftazidime 2.25 mg/0.1 ml), fortified topical antibiotics, and systemic ceftriaxone were given.

Her left eye eventually progressed to corneal perforation and was glued with tarsorrhaphy, as she refused all recommended procedures. She was eventually scheduled for left eye evisceration after hemodynamic stabilization. Unfortunately, the patient succumbed to infection.

Case 3

A 42-year-old Malay woman with underlying diabetes mellitus and bronchial asthma presented to the hospital with a sudden blurring of vision in the left eye for two days without any systemic symptoms. Visual acuity was 6/9 and PL on the right and left eye, respectively.

**Figure 6:**
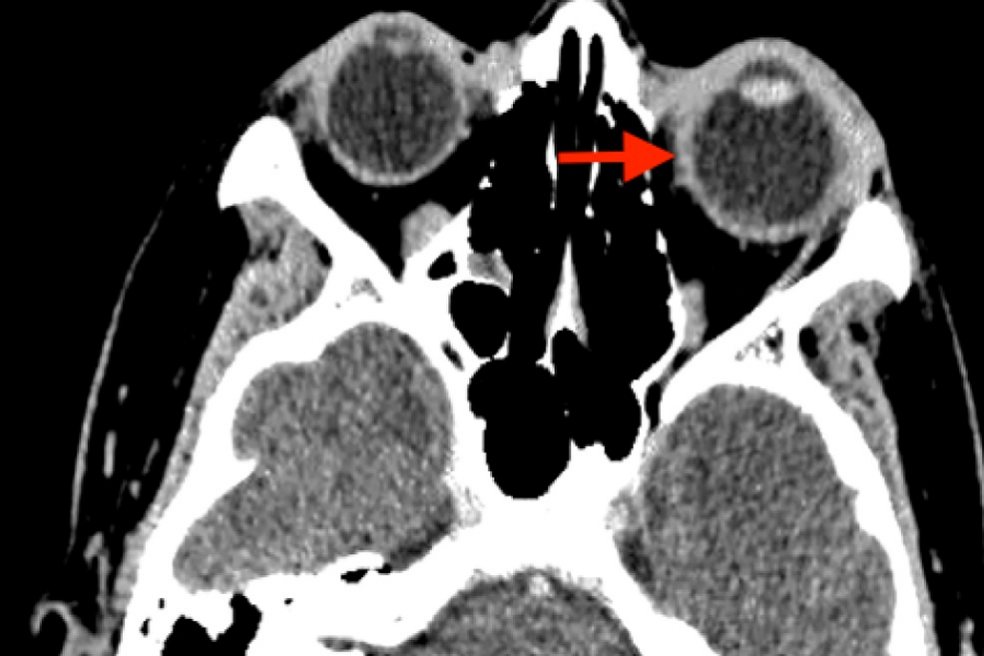
CECT brain/orbit shows left periorbital soft tissue thickening and enhancement suggestive of inflammatory/infective changes CECT: contrast-enhanced computed tomography

**Figure 7:**
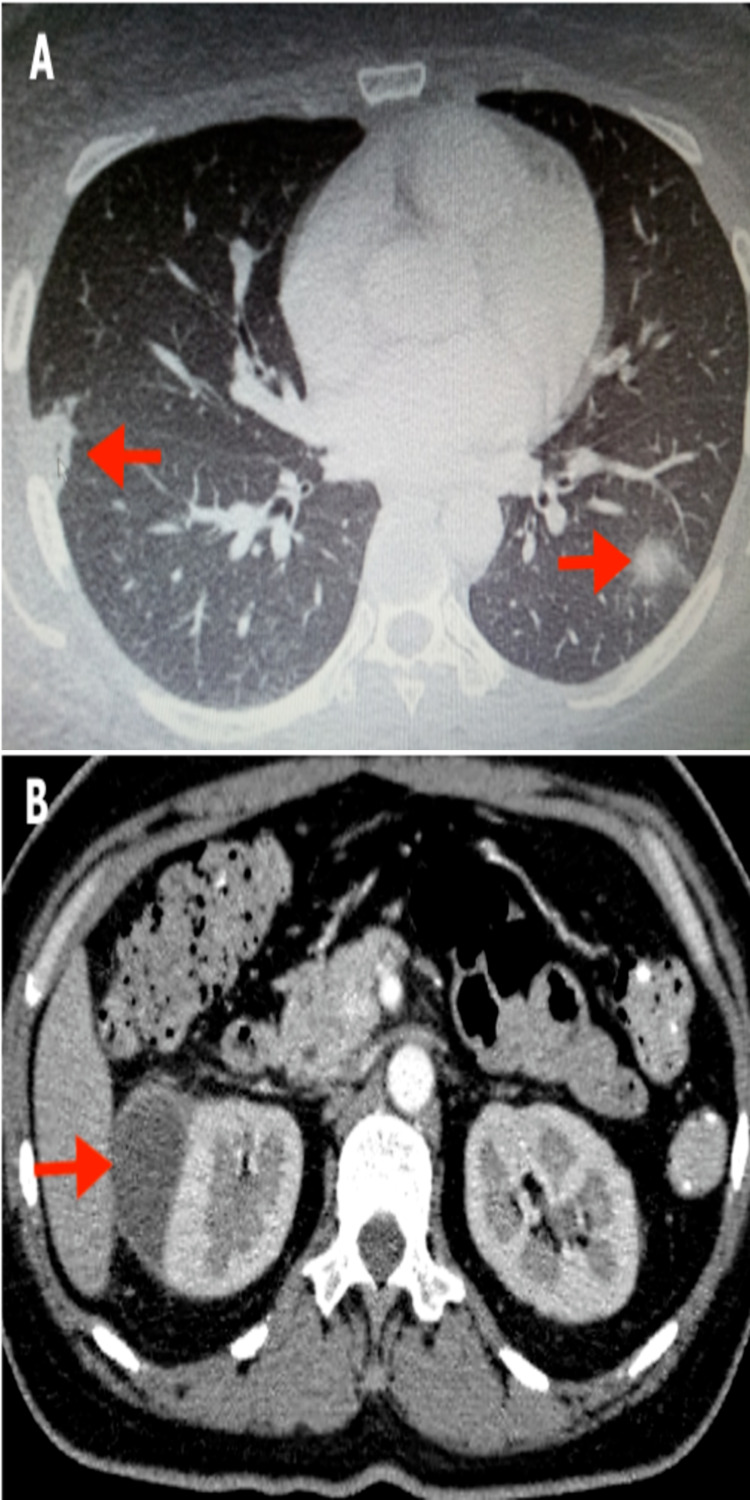
CECT thorax, abdomen, and pelvis shows (A) multiple cavitating lesions in both lungs suggestive of septic emboli; (B) right perirenal rim enhancing hypodense collection suggestive of an abscess CECT: contrast-enhanced computed tomography

RAPD was negative. Assessment of the left anterior segment revealed cells 4+ and 1 mm of hypopyon level. IOP of the left eye was 8 mmHg. There was no view of the fundus, however, ultrasound of the eye revealed the presence of loculation with a flat retina. Vitreous and blood culture showed no growth. *Klebsiella pneumoniae* was eventually detected from lung biopsy tissue culture. CECT brain and orbit showed left periorbital soft tissue thickening and enhancement suggestive of inflammatory/infective changes (Figure 6). CECT of the chest and renal system revealed multiple cavitating lung abscesses and renal collection (Figure 7). She was diagnosed with KPIS with a renal abscess and left endogenous endophthalmitis.

**Figure 8:**
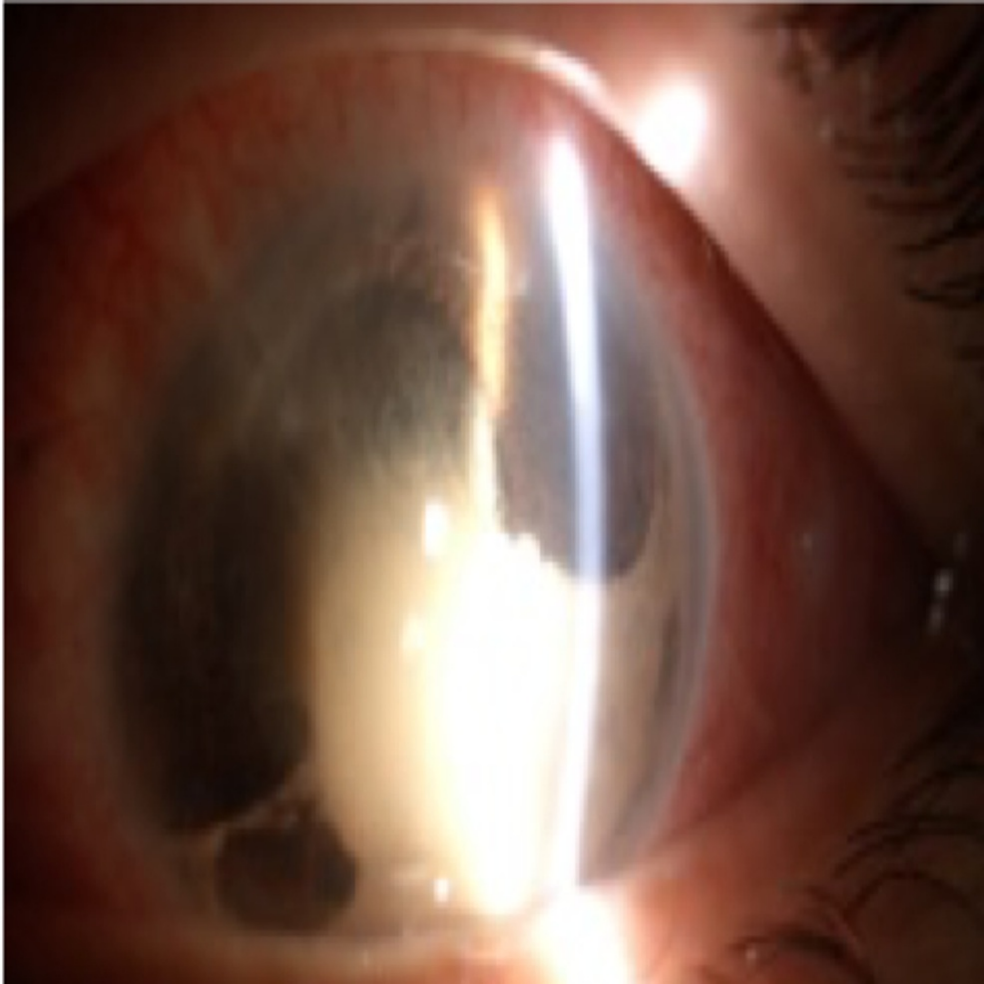
Contracting debris and fibrin following treatment.

Multiple intravitreal injections (vancomycin 1 mg/0.1 ml and ceftazidime 2.25 mg/0.1 ml) were performed. Systemic ceftriaxone was escalated to meropenem, as it has good coverage against most resistant gram-negative organisms. Fortified topical antibiotics were also commenced. The anterior segment inflammation reduced and the fibrin contracted following treatment (Figure 8).

She underwent a left vitrectomy, however, her left vision remained poor with nonperception to light (NPL) in all four quadrants.

## Discussion

*Klebsiella pneumoniae *is a well-known human nosocomial pathogen. *Klebsiella pneumoniae* is now the main cause of liver abscess reported in Hong Kong, Singapore, South Korea, and Taiwan [6]. In the past decades, the prevalence of *Klebsiella pneumoniae* invasive syndrome (KPIS) with extrahepatic complications has increased in Asia [7].

KPIS is a systemic manifestation caused by hypervirulent *Klebsiella pneumoniae* resulting in serious life-threatening community-acquired infections such as liver abscess, pneumonia, meningitis, and endophthalmitis [8-9]. In addition to the liver, it has the ability to metastatically spread (11%-80% of cases) to distant sites like lungs, eyes, kidneys, spleen, and bone marrow [8-10]. Diabetics are strongly predisposed to *Klebsiella* liver abscess and are at higher risk of metastatic spread of infection [6-11].

Endogenous Klebsiella endophthalmitis is a devastating ocular infection with most cases resulting in visual acuity of perception to light or worse being subjected to evisceration or enucleation [5].

All of our patients presented with mostly eye complaints rather than systemic symptoms. They were referred to the respective teams for co-management of systemic involvement upon further investigation. We would like to highlight that despite our patients presenting with eye symptoms as their chief complaint, we were alert to the possible systemic relationship and investigated and managed all of our patients ophthalmologically and systemically. Despite our maximum effort, one of our patients still succumbed to multiple organs dysfunction syndrome (MODS) due to the virulence of *Klebsiella pneumoniae*.

Two of our patients underwent evisceration, whereas one patient had NPL vision despite being given a comprehensive treatment of vitrectomy, multiple systemic antibiotics, intravitreal injections, and drainage of primary foci. Over the past three decades, the overall visual outcome in patients with EKE remains dismal despite early recognition and aggressive treatment. The overall rate of vision recovery surpassing counting fingers was around 22.64%-34% [12-13]. However, Hsieh MC et al. have found out that with early recognition, better outcomes were obtained with a good prognosis related to initial VA, female gender, and the number of intravitreal injections. Early intervention with pars plana vitrectomy did not change the visual outcome [13]. Our patients had delayed surgical intervention, as they were hemodynamically unstable and unfit for surgery.

## Conclusions

*Klebsiella pneumoniae *invasive syndrome (KPIS) is typically seen in East Asians with the risk factor of diabetes. We discussed three cases, which include risk factors and the prognosis and management of this debilitating condition. A high index of suspicion should be held on this demographic of patients. Our case discussion highlights the importance of early suspicion of systemic involvement in patients presenting with endogenous endophthalmitis. Prompt diagnosis, emergent radiographic evaluation, early adequate drainage, and appropriate treatment with antibiotics potentially improve survival and visual prognosis.
